# Pilot implementation of a monitoring and enforcement system for the International Code of Marketing of Breast‐milk Substitutes in Cambodia

**DOI:** 10.1111/mcn.12795

**Published:** 2019-06-21

**Authors:** Kroeun Hou, Mackenzie Green, Senveasna Chum, Christine Kim, Ame Stormer, Gary Mundy

**Affiliations:** ^1^ Helen Keller International New York New York USA; ^2^ Department of Epidemiology Columbia University, Mailman School of Public Health New York New York USA

**Keywords:** breastfeeding, breast‐milk substitute, Cambodia, International Code of Marketing of Breast‐milk Substitutes, monitoring system, promotion

## Abstract

Globally, monitoring and enforcement mechanisms for the World Health Organization's International Code of Marketing of Breast‐milk Substitutes are often lacking. The Cambodian government adopted the Code as the national standard in Sub‐Decree 133 on Marketing of Products for Infant and Young Child Feeding. Following the formation of a multisectoral Oversight Board and development of detailed guidance documents for the implementation and enforcement of Sub‐Decree 133, a 7‐month pilot was conducted in 2017 to trial a monitoring system in four urban areas of Cambodia. The pilot included training of monitors from the Ministries of Health and Commerce, screening for violations at retail locations and health facilities, testing reporting mechanisms, and taking actions against violators. During the pilot, 85 national‐ and subnational‐level monitors were trained, 392 site visits were made, 2,377 monitoring checklists were completed, and 11 warning letters were issued to violators. Half of the completed checklists (52.9%) indicated Code violations, yet monitors submitted zero violation reports. The pilot revealed modifications needed to the monitoring system: integrate monitor trainings into existing ministry training curricula for sustainability; enhance targeting of monitors for Sub‐Decree training; delineate clear roles and responsibilities for the national and subnational levels; simplify monitoring checklists and violation reports; and improve integration of monitoring activities into routine ministry operations. Before the Sub‐Decree 133 monitoring and enforcement system is implemented throughout Cambodia, revisions must be made to ensure the viability of this system. Challenges and lessons learned can also guide Code monitoring efforts being undertaken by other countries.

**Key messages**

- A system of monitoring for the Cambodian Sub‐Decree 133 on Marketing of Products for Infant and Young Child Feeding was piloted in Cambodia. Half (52.9%) of the completed monitoring checklists cited violations; yet national‐ and subnational‐level monitors filed zero violation and monthly monitoring reports.
- The pilot exposed gaps and challenges to the designed system. Each ministry involved needs to develop a clear, detailed work plan to integrate Sub‐Decree 133 monitoring into routine training, monitoring, and reporting services.
- Improved communication between stakeholders is needed to ensure that Sub‐Decree 133 monitoring trainings and activities occur seamlessly, and national and subnational monitors explicitly understand their designated roles within the monitoring system.
- Training modules and checklists for Sub‐Decree 133 monitoring should be revised to ensure that guidance on the monitoring process, methods, and materials is clearly explained, and ample time for teaching and activities is given.
- Clear follow‐up plans should be developed to track violators that received warnings and agreement letters. Repeat offenders should be given more stringent penalties.

## 1 INTRODUCTION

Scaling up of optimal breastfeeding practices could have the single largest impact on child mortality of any preventive intervention (Bhutta et al., 2013). Cambodia has made significant progress improving its rate of exclusive breastfeeding from 11% in 2000 to 74% in 2010 (National Institute of Statistics [NIS], Directorate General for Health, & ICF Macro, 2011); however, the 2014 Cambodia Demographic Health Survey found that exclusive breastfeeding and continued breastfeeding at 2 years of age declined to 65% and 37%, respectively (NIS, Directorate General for Health, & ICF International, 2015). In 2010, bottle‐feeding for infants less than 6 months old increased to 14%, from 3% in 2000, and reached more than 30% for infants less than 12 months (NIS, Directorate General for Health, & ICF Macro, 2011). The use of breast‐milk substitutes (BMS) has also become more common over the last two decades (National Institute of Public Health/National Institute of Statistics [NIPH/NIS], & ORC Macro, 2006; NIS, Directorate General for Health, & ICF Macro, 2011), with a 2014 survey finding 41% of women surveyed in Phnom Penh provided BMS to their child aged 0–24 months (Pries et al., 2016).

Commercial promotions for BMS are increasingly prevalent in Cambodia. A 2014 survey found 86% of mothers of children less than 24 months old had observed commercial promotions for BMS and 19% observed BMS product logos on health facility equipment (Pries et al., 2016). Reviews of television advertisements airing in Cambodia indicate increasing BMS promotions: approximately 3 hours per month of BMS advertisement time on television in 2013–2014, growing to 20 hours per month of advertisement time in 2015–2016 (Alive & Thrive, 2016; Champeny et al., 2019). Commercial promotions of BMS products contribute to suboptimal breastfeeding practices (Piwoz & Huffman, 2015; Rollins et al., 2016). Mothers with low education or access to resources are also more susceptible to BMS promotions and the risks BMS use may pose to their children (Barennes, Slesak, Goyet, Aaron, & Srour, 2016).

In response to documented, unethical marketing activities by BMS manufacturers, the World Health Organization (WHO) developed the *International Code of Marketing of Breast‐milk Substitutes* (the Code; WHO, 1981) and subsequent resolutions. Although the Code has been in existence for more than 35 years, WHO reports that few countries have a monitoring mechanism in place to regulate promotional practices, and of those, few are fully functional (WHO, UNICEF, & International Baby Food Action Network [IBFAN], 2016). Only 32 countries report having a formal monitoring system in place and less than a quarter of countries with a monitoring mechanism publish results of their monitoring exercises. Just six countries report having dedicated budgets or funding for monitoring and enforcement.

In 2005, the Cambodian government adopted many provisions of the Code as national policy in the Cambodian *Sub‐Decree on Marketing of Products for Infant and Young Child Feeding* (Sub‐Decree 133), to support breastfeeding by restricting the promotion of BMS marketed for children less than 2 years of age, unless approved by the Ministry of Health (MOH; Royal Government of Cambodia, 2005). The subsequent Joint Proclamation (*Prakas*) 061 on the *Marketing of Products for Infant and Young Child Feeding* was intended to operationalize implementation of Sub‐Decree 133 (MOH, 2007). However, no follow‐up action was taken to implement procedures or establish an ongoing monitoring system that would identify and address Sub‐Decree 133 violations.

In 2014, a multisectoral Oversight Board was created to spur action and improve the monitoring and enforcement of Sub‐Decree 133. The MOH was designated to lead the Oversight Board, and the three additional ministries tasked with enacting Sub‐Decree 133—Commerce, Information, and Industry and Handicraft—were each represented by their Secretary of State. The Oversight Board was designed with two executing arms: (a) the Control Committee and (b) the Executive Working Group (EWG). The Control Committee reviews and approves all labels and packaging for infant and young child feeding (IYCF) products before they are imported and/or sold in Cambodia, along with approving all marketing materials and promotional activities before use. The EWG, composed of members of the four ministries, oversees monitoring compliance and enforcement, including receiving, reviewing, and acting on violation reports submitted by the ministries, civil society, and regular citizens.

After creation in 2015, the EWG recognized a need for the explicit definition of responsibilities in order to operationalize the Sub‐Decree. In 2015, the EWG developed *Implementation Guidelines*, further detailing the roles of the four ministries at the national and subnational level for the implementation, monitoring, and enforcement of Sub‐Decree 133 (MOH, 2015a). Clear *Terms of Reference* for the Oversight Board, EWG, and Control Committee were also developed (MOH, 2015b). Both guidance documents, endorsed by the Oversight Board in late 2015, placed an emphasis on integrating monitoring activities into the existing, routine responsibilities of the four ministries, to reduce the need for additional financial and human resources and to increase sustainability and continuity of monitoring. To facilitate the monitoring activities of national and subnational officers, checklists from NetCode, the Network for Global Monitoring and Support for Implementation of the International Code of Marketing of Breast‐milk Substitutes and Subsequent Relevant World Health Assembly Resolutions (WHO & UNICEF, 2017a), were adapted through a multisectoral process to adhere to the scope of Sub‐Decree 133 and Joint Proclamation 061. Checklists were translated into Khmer and endorsed by the EWG.

With the new multisectoral oversight bodies in place and the guidance materials endorsed, the government, with support from Helen Keller International (HKI), WHO, and UNICEF, set out to pilot the new Sub‐Decree 133 monitoring and enforcement system and to propose revisions, before implementing the system throughout Cambodia. This paper describes the pilot implementation of the monitoring and enforcement system and the observed shortcomings and successes.

## 2 MATERIALS AND METHODS

### 2.1 Pilot study design

This study was designed to trial the newly developed monitoring and enforcement system that entailed training of monitors, screening for violations, reporting mechanisms, and taking actions against violators. The pilot focused on the monitoring process once products entered the market for sale, including monitoring promotions and product labels at points‐of‐sale and promotions at health facilities. The pilot did not trial the system prior to product importation/manufacture or sale.

Four urban locations were selected for this pilot: Battambang, Siem Reap, Sihanouk, and Phnom Penh. These locations were chosen for their availability of BMS products, accessibility of large shops/markets, and active health facilities. The pilot was implemented from January to August 2017 and examined four components: (a) training; (b) completion of checklists and reports; (c) provision of feedback to stores and health facilities; and (d) actions taken against violators.

The MOH and Ministry of Commerce at the national and subnational levels participated in the pilot. The trial did not include the Ministry of Industry and Handicraft and the Ministry of Information even though Sub‐Decree 133 outlines monitoring responsibilities for them once products enter the market for sale. The Ministry of Industry and Handicraft is called to review the labelling and packaging of locally produced IYCF products; yet, at the time of the pilot, there were no local manufacturers of IYCF products. The Sub‐Decree also calls for the monitoring of product promotions on all media platforms, including television, newspaper, radio, and online; however, discussion is still ongoing as to whether this responsibility falls to the Ministry of Information or another ministry.

### 2.2 Pilot preparation

Preparation activities were led by the National Nutrition Program (NNP) in the MOH, the Department of Drug and Food Safety (DDF) in the MOH, and CAMCONTROL in the Ministry of Commerce. In collaboration with HKI, WHO, and UNICEF, the departments established a pilot joint work plan and timeline and discussed monitoring roles to ensure no overlap between ministries. The joint work plan detailed each department's responsibilities, key activities, expected outputs, and resources allocated for each activity. In the work plan, each department agreed to visit 30 sites per urban location, or 360 sites in total across all ministries and locations. This number was based on feasibility, capacity, and their pre‐existing routine monitoring schedules or related activities.

### 2.3 Role of ministries in pilot monitoring system

The roles and responsibilities for each participating ministry and department were outlined in the joint work plan according to the *Implementation Guidelines* of Sub‐Decree 133. Monitoring and reporting activities during the pilot were to be conducted in addition to their ongoing responsibilities and were as follows.

#### 2.3.1 Ministry of Commerce, Department of CAMCONTROL

CAMCONTROL subnational officers were responsible for monitoring during their routine activities at supermarkets, minimarts, and small shops for point‐of‐sale promotions and to review IYCF product labels for violations. These officers were authorized CAMCONTROL inspectors who already carried out regular ministry inspections of retail locations. The adapted checklists guided their Sub‐Decree 133 monitoring, and any suspected violations were to be reported directly to the EWG by the inspector. Each month of the pilot, CAMCONTROL was responsible for submitting a summary report to the EWG of all national and subnational monitoring activities.

#### 2.3.2 Ministry of Health, Department of Drug and Food Safety

DDF's national and subnational level officers were to monitor promotional activities at pharmacies and specialty baby shops and to review product labels for violations. Similar to CAMCONTROL, DDF monitors were authorized DDF inspectors who routinely visited retail locations to ensure compliance with government regulations, and under the pilot were to integrate the use of Sub‐Decree 133 checklists into their ongoing inspections. Subnational monitors were required to submit violation reports directly to the EWG as well as monthly reports on their inspection.

#### 2.3.3 Ministry of Health, National Nutrition Program

NNP was to monitor advertising and promotional activities in health facilities during routine facility visits made by their subnational Nutrition Focal Points to support nutrition‐related maternal child health services. The Nutrition Focal Points were to monitor product promotions in Cambodia's health care system including the distribution of free or subsidized supplies of BMS. They were also tasked with submitting monthly reports on monitoring activities and any violation reports to the EWG.

### 2.4 Pilot study tools

#### 2.4.1 Monitoring checklists

Three checklists adapted from NetCode protocol tools were used during the pilot (WHO & UNICEF, 2017a). Checklists guided monitoring of (a) labels and packaging of products (Appendix S1); (b) point‐of‐sale promotions (Appendix S2); and (c) health facility promotional materials and activities (Appendix S3). The *Implementation Guidelines* define promotion as “advertising, sampling, or any other activity to encourage or induce the purchase of a product” (MOH, 2015a). The detailed checklists provided a thorough evaluation of retail locations, health facilities, and package labels to identify any violation of Sub‐Decree 133, including inspecting labels and promotional materials for the MOH stamp indicating approval from the Control Committee. One point‐of‐sale checklist and one health facility checklist were to be completed per respective location. Labelling checklists should have been completed for each relevant product found for sale at the retail locations.

#### 2.4.2 Sub‐Decree 133 violation reports

The pilot used the Sub‐Decree 133 violation reporting form from the *Implementation Guidelines* (Appendix S4; MOH, 2015a). The form captured information on (a) description of the violation; (b) date and location of the violation; (c) company and brand in the violation; (d) type of product in the violation (e.g., infant formula or follow‐up formula); (e) type of violation (e.g., promotion in shop, free sample, or inadequate labelling); and (f) name and contact information of the person reporting the violations. The form also requests submission of photos or other evidence of the violation.

In the pilot, all national and subnational monitors finding violations during their activities were required to submit a completed form for each violation observed directly to the Secretaries of the EWG.

#### 2.4.3 Monthly monitoring reports

National and subnational monitors were to submit monthly reports summarizing their monitoring activities to their ministry's designated focal person at the national level, who then sent the reports to the EWG. Monthly reporting forms were taken from the *Implementation Guidelines* (MOH, 2015a) and detailed the following: (a) date of monitoring; (b) type of monitoring (e.g., point‐of‐sale or health facility); (c) location of monitoring, including city/district and name of site/shop/health facility; (d) key findings from monitoring (e.g., labels not compliant); (e) list of noncompliant products; and (f) actions taken.

### 2.5 National and subnational monitor trainings

A two‐day consultative training workshop was held in September 2016 to develop training materials and collectively decide on the training methods and logistics at both the national and subnational level. Participants included EWG members, national‐level master trainers in nutrition, ministry officers, and representatives from HKI and WHO.

A series of two‐day trainings for national and subnational monitors from CAMCONTROL, NNP, and DDF were held in Siem Reap and Phnom Penh in November 2016 and January 2017. Trainings were led by the nutrition master trainers and EWG members. Subnational monitors attended from the four pilot locations, chosen to participate by their respective departments for their regular roles as authorized inspectors or Nutrition Focal Points. The trainings covered Sub‐Decree 133 and monitoring procedures, such as completing and submitting checklists, violation reports, and monitoring reports. Fifteen‐question pre‐tests/post‐tests were administered to participants to assess mastery of the training content.

### 2.6 Monitoring visits by national and subnational teams

Monitoring activities were piloted from January to August 2017. The subnational monitoring teams from CAMCONTROL, DDF, and NNP were to implement their respective monitoring procedures as part of their routine responsibilities, including completion of checklists, consolidation of checklist information into monthly reports, and submission of monthly reports to their provincial departments and EWG. Monitoring locations were chosen purposely and informed by routine activities of each department. Stores selling BMS products were selected from the schedule of stores to be visited by CAMCONTROL and DDF monitors during that timeframe. Health facilities were selected from the routine visit schedule and those offering maternal child health services, with priority placed on higher level and/or busier facilities. Locations were not informed ahead of time when monitoring would occur.

National teams from CAMONTROL, DDF, and NNP were responsible for three rounds of spot checks on adherence to monitoring procedures and providing technical support to the subnational monitoring teams. During these spot checks, national officers accompanied the subnational monitors to the retail locations or health facilities, observing procedures, identifying problems, and offering constructive feedback to the subnational teams. These teams worked independently and coordinated schedules to avoid overlap in the four areas. The purpose for each round of spot check was:

Round 1: Round 1 was conducted in March–April 2017. The purpose of this initial period was to ensure that all monitoring activities had begun.
Round 2: Round 2 occurred May–June 2017, to assess the continuation of monitoring activities and to begin to identify challenges and limitations of the system. EWG members accompanied teams on some visits, and key findings were shared with the EWG and Oversight Board.
Round 3: Round 3 of pilot activities took place in July–August 2017. The aim was to gather information on monitoring and enforcement experiences, including monitoring practices, use of checklists and monthly forms, violation reports, and EWG feedback.

### 2.7 Operation of EWG and Oversight Board

During the pilot, the EWG was to receive violation reports and the monthly monitoring reports from each ministry/department. As per the *Implementation Guidelines* and *Terms of Reference*, the EWG was to meet once a month to review monitoring activities, suspected violations, and to issue action on violations. The Oversight Board was to meet once every three months.

### 2.8 Data management and analysis

With support from a WHO consultant, an Excel database system was established at DDF to track the completed checklists for all monitoring activities from the pilot period. After each round of field visits, the two ministries submitted their completed checklists to DDF for entry. Based on the *Implementation Guidelines*, the checklists typically should remain at the subnational level where they are used, but to facilitate assessment for this pilot, the checklists were shared with the national level DDF and entered by the WHO consultant and DDF staff.

To assess implementation of the system during the pilot and to develop recommendations for improvement, a number of resources were reviewed and discussions held with national and subnational monitors and officials. The checklist database was analysed in Excel to calculate the number and type of monitoring visits made, checklists completed, and violations detected and reported. A WHO rapid review of the use of the checklists, which included focus group discussions with subnational monitors and key informant interviews with national ministry officials, was reviewed (Iellamo, 2017). Numerous official and unofficial meetings were held throughout the course of the pilot with EWG members, Oversight Board members, the nutrition master trainers, the national teams who carried out the spot checks, and partners including HKI, WHO, and UNICEF. A final consensus meeting was held by the Chair of the EWG in January 2018 and attended by all involved in the pilot implementation, where experiences, lessons learned, and next steps for the ministries and EWG were discussed.

## 3 RESULTS

### 3.1 Sub‐Decree 133 monitoring trainings

Table 1 displays the participant turnout from the relevant ministries at the national‐ and subnational‐level monitor trainings. In total, 16 monitors were expected to participate from the national level and 20 from the subnational level; however, attendance was higher than expected. From the national level, 33 attended and 52 from the subnational level. The average pre‐test score across the trainings was 8.0 out of 15, and the average post‐test score was 12.4 out of 15.

**Table 1:** Participant turnout at Sub‐Decree 133 monitoring system trainings

Ministry (Department)	National‐level monitors		Subnational‐level monitors	
	Anticipated (*n*)	Actual (*n*)	Anticipated (*n*)	Actual (*n*)
Health (NNP and DDF)	8	17	10	38
Commerce (CAMCONTROL)	8	16	10	14
Total	16	33	20	52

*Note*. DDF: Department of Drug and Food Safety; NNP: National Nutrition Program.

### 3.2 Monitoring in the four provinces

Table 2 presents the number of monitoring visits made during the pilot study. Although it was proposed that a total of 360 visits be made over the pilot, 392 visits were made in total. It was possible for a site to have been visited more than once during the pilot, as sites were selected and visited based on the routine activities and schedules of DDF, CAMCONTROL, and NNP. DDF monitors made 128 site visits to points‐of‐sale, with 97 pharmacy visits and 31 baby shop visits. CAMCONTROL monitors attended 106 site visits: 13 supermarket visits and 93 minimarts/small shop visits. NNP made 158 monitoring visits to health facilities.

**Table 2:** Number of monitoring visits made during the pilot

Department	Anticipated number of visits (*n*)	Type of site visited	Actual number of visits (*n*)
Department of Drug and Food Safety	120	Pharmacy	97
		Baby shop	31
CAMCONTROL	120	Supermarket	13
		Small shop/minimart	93
National Nutrition Program	120	Health facility	158
Total	360	—	392

### 3.3 Monitors' adherence to Sub‐Decree 133 monitoring and reporting guidelines

Table 3 displays the completion of checklists by monitors from DDF, CAMCONTROL, and NNP over the pilot period. In total, 2,377 checklists were completed, with 2,169 for labels, 50 for point‐of‐sale promotions, and 158 for health facility promotions. Half of all checklists (52.9%) identified noncompliance with Sub‐Decree 133. Point‐of‐sale checklists were more likely to indicate violations (86.0%) than label checklists (53.0%) and health facility checklists (40.5%). Of the 43 point‐of‐sale violations, 9.3% (4) were for free samples or gifts, 11.6% (5) for product discounts, and 79.1% (34) for display or printed material promotions. Of the 64 violations observed in health facilities, 4.7% (3) were promotions for free samples or gifts and 95.3% (61) for displays or printed materials.

**Table 3:** Monitoring and adherence to Sub‐Decree 133 checklist guidelines

Department	Monitoring location	Type of checklist	Number of checklists (*n*)		
			Completed	Compliant	Noncompliant
Department of Drug and Food Safety	Pharmacy	POS	6	2	4
		Label	519	263	256
	Baby shop	POS	12	0	12
		Label	433	181	252
CAMCONTROL	Supermarket	POS	4	4	0
		Label	449	207	242
	Small shop/minimart	POS	28	1	27
		Label	768	368	400
National Nutrition Program	Health facility	Facility	158	94	64
Total	Retail location	POS	50	7	43
	Retail location	Label	2,169	1,019	1,150
	Health facility	Facility	158	94	64

*Note*. POS: point‐of‐sale.

There was no evidence of subnational monitors submitting monthly reports summarizing their monitoring activities to their respective ministry nor of the ministry‐designed focal person at the national level reporting to the EWG of monthly activities (Table 4). Additionally, no violation reports were generated and submitted to the EWG by either the subnational or national monitoring teams.

**Table 4:** Monitoring and adherence to Sub‐Decree 133 reporting guidelines

Monitoring and enforcement task	Number (*n*)
Monthly monitoring reports	
Summary reports submitted by ministries	0
Violation reports	
Violation reports submitted by national level	0
Violation reports submitted by subnational level	0
Actions against violators	
Warning letter issued to companies/distributors	11
Warning letter issued to point‐of‐sale	0
Warning letter issued to health facility	0
Financial penalty levied	0

### 3.4 Oversight Board, EWG, and actions taken against violators

Over the pilot period, the EWG met one time and the Oversight Board did not meet at all. By August 2017, the EWG issued a total of 11 warning and agreement letters to companies/distributors who were found to have label/package violations during the pilot monitoring (Table 4). Warning letters indicated that the company/distributor had violated Sub‐Decree 133, and by signing, the company agreed to resolve the violation within a specified period of time (typically 2–3 months). No warning letters were issued for promotional activities found at points‐of‐sale or health facilities; monitoring teams instead chose to verbally discuss the violation with store owners/managers and facility management.

## 4 DISCUSSION

This pilot provides important understanding of the initial implementation of a routine monitoring and enforcement system to enforce the Code and protect breastfeeding practices. Cambodia is the first country to pilot a monitoring system utilizing adapted versions of NetCode's Code monitoring tools, and this is one of few descriptions of the early stage of operationalizing national legislation into a functioning monitoring system (WHO & UNICEF, 2017b; WHO, UNICEF, & IBFAN, 2016). Over the implementation period, nearly 400 monitoring visits were made guided by these tools, with a significant number of violations detected and warning letters issued to violators. The pilot highlighted the successful elements of routine monitoring and enforcement of Sub‐Decree 133, and also the challenges that still need to be addressed before full implementation throughout Cambodia. These successes and challenges have informed recommendations for future implementation, both for the Royal Government of Cambodia and other countries seeking to enact monitoring and enforcement systems.

### 4.1 Training of monitoring personnel

Far more monitors attended the trainings than originally planned. According to the ministries, the discrepancy in the turnout may have occurred because DDF and CAMCONTROL seized this opportunity to train their entire staff on the monitoring and enforcement curriculum. Positively, this reflects political commitment and interest in enforcing Sub‐Decree 133 at all levels; however, the larger‐than‐anticipated attendance resulted in staff without monitoring responsibilities being trained and having to add extra training sessions in January 2017 to fully reach the intended targets. Improved communication, coordination, and targeting is recommended to save resources.

After the national and subnational level trainings, trainers noted that significant refinement was required in the training materials, activities, and time allocation. During the pilot, subnational monitors also conveyed that they did not feel fully competent in their designated roles (Iellamo, 2017). Proposed future modifications to the training include adding more content on the detailed steps of the monitoring process, providing participants with additional time to practice filling in checklists and reporting forms, and inserting more opportunities to identify the differences between compliance and noncompliance, especially for inspectors visiting points‐of‐sale.

The national and subnational level trainings in the pilot were both financially and technically supported by development partners, with consideration that the government would take responsibility in providing resources when the system is eventually rolled out in other provinces. Trainings were also stand‐alone, although several of the ministries have routine, scheduled trainings for their cadre of inspectors, including DDF and CAMCONTROL. To improve sustainability of monitoring, we recommend that Sub‐Decree 133 training content should be fully integrated into each ministry's routine training system.

### 4.2 Monitoring tools and process

Monitors made 392 visits during the pilot study; however, the implementation of these visits did not fully adhere to Sub‐Decree 133's *Implementation Guidelines*. Monitoring activities only occurred during the spot checks by national teams. These spot checks were supported by partners, which may explain why independent, subnational monitoring did not happened as planned. Although integrating Sub‐Decree monitoring into routine responsibilities should reduce the need for resources, subnational and national staff in all ministries and departments voiced concerns during the rapid review (Iellamo, 2017) and consensus meeting about the financial and human resources required to continually conduct monitoring activities. The EWG called on the ministries to reexamine and improve their integration efforts and allocation of resources, but the challenge has not yet been fully resolved. The lack of financial support remains a recurring issue for countries making a concerted effort to implement the Code. According to the WHO's *2011 Status Report*, countries identified that the lack of appropriate funding and capacity made it difficult to effectively conduct monitoring activities (WHO, 2013). Fully integrating BMS monitoring into functioning routine inspection systems can be a solution to maximizing available resources, but in instances where systems do not exist or are not well‐functioning, alternative solutions may be needed, such as integrating into health facility accreditation or other maternal, infant, and young children health initiatives (WHO & UNICEF, 2017b; WHO & UNICEF, 2018). Governments will also need to prioritize the allocation of funds for their monitoring and enforcement system to function at all levels.

National and subnational monitors frequently mentioned that the multipage monitoring checklists should be simplified because they were long and redundant. Although the forms were developed to guide thorough inspections, if the length inhibits monitoring or increases time and human resource needs, then revisions must be considered. CAMCONTROL, DDF, and NNP have all requested that the checklists be reorganized to prioritize key indicators of BMS violations including label translations, product/promotion approval from the Control Committee, and point‐of‐sale/facility promotions of BMS. For ministry departments that already have routine inspection procedures and checklists, such as CAMCONTROL and DDF, key indicators could be integrated or streamlined into their tools to encourage consistent use. In cases where existing tools are not available or the underlying system requires strengthening, simple stand‐alone tools may be the best approach.

### 4.3 Reporting of monthly activities and violations

Over the pilot period, none of the monitoring teams submitted violation reports or monthly summary reports to the EWG. In informal discussions and during the rapid review, subnational monitors reported confusion over their roles and responsibilities and limited time and resources for the extra steps of reporting violations to the national‐level monitors (Iellamo, 2017). Subnational monitors did not submit any reports because they were waiting on national monitors to follow‐up first. More notably, subnational monitors reported that their responsibilities requiring them to directly report to the national level were at odds with the structural hierarchy of their ministries; low‐level officers do not report directly to high‐level officials. The integration of responsibilities must also follow the existing flow of information and reporting within the ministries. Close coordination with ministry staff at all levels is vital to understanding their operations and finding opportunities to build monitoring into their existing system.

There is evidence that a multisectoral approach to ensuring compliance with the Code can work. Through a comprehensive monitoring strategy between government, UNICEF, and non‐governmental organizations, the Indian state of Assam increased exclusive breastfeeding from 29% in 1999 to 63% in 2016 (Barennes et al., 2016). Proper coordination between the government and civil society organizations is pivotal to ensure that companies are in compliance with national Code laws (Barennes et al., 2016). In Cambodia, civil society groups do not have a formal role in Sub‐Decree 133 monitoring and enforcement, but they are encouraged to help by reporting any violations they observe and urging the government to enforce the Sub‐Decree. The Scaling Up Nutrition Civil Society Alliance Cambodia has held trainings for their members on Sub‐Decree 133, and since the pilot, a number of members have submitted violation reports to the EWG. Additional engagement and education of civil society is needed in Cambodia, as a multisectoral and collaborative approach provides a more comprehensive and accountable system for monitoring and enforcement of Sub‐Decree 133.

### 4.4 EWG review and action taken against violators

According to its *Terms of Reference*, the Oversight Board should have conducted quarterly meetings to review the system's challenges and agree on appropriate solutions to be taken (MOH, 2015b). However, the Oversight Board did not hold any formal meetings between January and August 2017. The Chair of EWG and its members had several communications with the Chair of the Oversight Board; however, they failed to hold a formal meeting (Iellamo, 2017). The *Terms of Reference* also state that the EWG shall conduct monthly meetings to review monitoring activity results, to address issues regarding the ministries' responsibilities, and to discuss challenges and appropriate solutions (MOH, 2015b). Despite this, the EWG held only one meeting during the pilot, in April 2017. Both the Oversight Board and EWG members are high‐ranking officials, and the ministries reported difficulty in coordinating schedules to adhere to the required meeting frequencies. At DDF and NNP recommendation during the consensus meeting, the EWG has made modifications to hold bimonthly meetings instead. The Oversight Board should consider similar modifications, with the caveat to call meetings if urgent issues arise. Additionally, collaboration between the EWG and the Control Committee needs strengthening, which can be instituted with the regular sharing of information and feedback regarding monitoring activities.

Warning and agreement letters were sent to 11 companies found in violation of Sub‐Decree 133 during the pilot, requiring that they comply by creating proper product labels. The government's issuance of these letters was considered a “soft approach” against violators (Iellamo, 2017) and may have been taken to build awareness of Sub‐Decree 133, its standards, and that the government is intent on taking action. At the time of the pilot, Cambodia did not have any punitive measures in place for violators of Sub‐Decree 133. During the three rounds of spot checks, it was also observed that monitors discussed violations they found with health facility staff and shop owners. Although this may provide an opportunity to learn and correct actions, so far, shop owners have been unwilling to remove products that are noncompliant (Iellamo, 2017).

The MOH and Ministry of Commerce will need to review the long‐term impact of their soft approach and determine if it is having the intended consequence of reducing violations. There is still a need to conduct follow‐up meetings with the companies and distributors that received warning and agreement letters to see if they have complied. Furthermore, it is recommended that the Cambodian government take additional steps against violators that do not comply with the mandates outlined in these letters. The Oversight Board and EWG should consider harsher punishments for Sub‐Decree violators, especially repeat offenders. The use of financial penalties could be one mechanism to offset the financial costs of monitoring if the fines are reinvested into the system, which has been used successfully to regulate tobacco promotion (Ioannidis, Henriksen, & Prochaska, 2013).

National policies to restrict the marketing and promotion of BMS without enforcement are ineffectual (Barennes et al., 2016). Article 11.2 of the Code places the responsibility of monitoring legislation on governments (WHO, 1981). Use of ongoing, routine monitoring systems can lead to immediate results, especially if the system involves inspectors from national agencies, such as food, drug, and customs, who already have the power to control and regulate products and advertising (WHO & UNICEF, 2017b). Functioning national monitoring systems can also serve as a deterrent, notifying manufacturers and distributors that compliance is being examined and violators will be held accountable for their actions (WHO & UNICEF, 2017b).

## 5 CONCLUSION

The pilot implementation of Sub‐Decree 133's monitoring and enforcement system in Cambodia presented successes, challenges, and recommendations for moving ahead. Political commitment to abiding by Sub‐Decree 133's guidelines was apparent; however, changes are needed to strengthen and ensure the seamless execution of the monitoring system before it is further enacted throughout Cambodia.

Cambodia should develop an action plan for the nationwide implementation of Sub‐Decree 133's monitoring and enforcement system, based on experiences and lessons learned from the pilot. This action plan needs to include a refined training system for national and subnational monitors, with improved communication and planning to allow facilitators to execute the training curricula effectively and with the appropriate staff. National and subnational monitors should have a clear understanding of their assigned roles and tasks within the monitoring and enforcement system before beginning their inspections. Sub‐Decree training should be incorporated into routine ministry operations, including booster trainings to refresh monitors' familiarity with violations.

Monitoring checklist could be shortened and refined to focus on key BMS violation indicators, enabling them to be better integrated into the ministries' routine activities or to simplify stand‐alone monitoring. This will encourage subnational monitors to complete checklists, especially as they are expected to act without the supervision of national monitors. It is vital that the enforcement system's activities become a consistent part of the ministries' routine operations, either as integrated activities or with alternative approaches that account for capacity and resources, to ensure the sustainability of the system. A follow‐up plan should be developed to track violators who received warning and agreement letters. If violators remain noncompliant, Cambodia should consider taking a more strident approach against Sub‐Decree 133 offenders, and those with repeat violations should be given more punitive penalties.

Finally, the EWG and the Oversight Board should ensure routine meetings are held to discuss the system's current achievements, difficulties, and solutions. With continued dedication and flexibility on behalf of the Royal Government of Cambodia, along with enhanced multisectoral involvement, a routine monitoring and enforcement system for Sub‐Decree 133 can successfully regulate promotion of BMS products and protect and promote breastfeeding throughout Cambodia.

## CONFLICTS OF INTEREST

The authors declare that they have no conflicts of interest.

## CONTRIBUTIONS

HK and MG conceptualized the pilot design. HK and CS supported the implementation of the pilot. HK prepared the manuscript, with support from MG and CK. All authors reviewed and provided significant input on the final article.

## Supporting information

Data S1. Pilot checklist for monitoring labels and packaging of products

Data S2. Pilot checklist for monitoring promotions at the point‐of‐sale

Data S3. Pilot checklist for monitoring at health facilities

Data S4. Pilot Violation Reporting Form
