# Supplementary material for: Pilot implementation of a monitoring and enforcement system for the International Code of Marketing of Breast‐milk Substitutes in Cambodia
Source: Matern Child Nutr. 2019 Jun 21;15(Suppl 4):e12795. doi: 10.1111/mcn.12795 (PMC6618142; doi:10.1111/mcn.12795)
Supplement: Supplementary file 1 — Data S1. Pilot checklist for monitoring labels and packaging of products [file MCN-15-e12795-s001.docx]

Appendix I: Pilot checklist for monitoring labels and packaging of products

**CHECKLIST FOR LABELS AND PACKAGING**

**FOR THE MARKETING OF PRODUCTS FOR INFANT AND YOUNG CHILD FEEDING**

| **General Information** | | |
| --- | --- | --- |
| Name of Province: | | |
| Name of City/District: | | |
| Name of Commune: | | |
| Name of Store/Shop/Pharmacy/place: | | |
| 1. Product types mentioned in the material (CHECK the one that APPLIES)   🞏 Infant formula (0+ months)  🞏 Follow up/on formula (6 + months)  🞏 Growing up milk (12 + months)  🞏 Any other milk for children 0-24 months  🞏 Any other food or liquid for children 0-24 months  🞏 Commercial complementary food or liquid (6+months)  🞏 Feeding bottles, teats, pacifiers  🞏 Similar Product | | |
| 1. Is there approval from the Control Committee?   *Enter the registration number of the MOH*:____________________ | 🞏 Yes 🞏 No |  |
| **If you answered NO, then STOP the inspection and report.**  ***If you answered* YES, then please continue the inspection.** | | |
| ***Statements of potential violations for all labels***  ***(check YES if present, NO if not present)*** | **CHECK THE ANSWER** | **YOU NEED TO REPORT IF THE ANSWER IS** |
| 1. Product information is printed on the container or a well-attached label? | 🞏 Yes 🞏 No | NO |
| 1. Is the language of the label in Khmer and easily readable? | 🞏 Yes 🞏 No | NO |
| 1. Is there a recommended age of introduction? *(If NO, go to 7)* | 🞏 Yes 🞏 No | NO |
| 1. Is the recommended age of introduction appropriate for that product? | 🞏 Yes 🞏 No | NO |
| 1. Does it contain any health and nutrition claims that suggest the effects of the product on the child?   *Specify the claim:*____________________________________________  *__________________________________________________________* | 🞏 Yes 🞏 No | YES |
| 1. Does it include an invitation to the mothers to contact the company? *Specify the invitation:*________________________________________ | 🞏 Yes 🞏 No | YES |
| 1. Does it include a list of the ingredients? | 🞏 Yes 🞏 No | NO |
| 1. Does it display nutritional composition of the product? | 🞏 Yes 🞏 No | NO |
| 1. Does it contain storage instructions? | 🞏 Yes 🞏 No | NO |
| 1. Does it contain the lot/batch number? | 🞏 Yes 🞏 No | NO |
| 1. Does it show the date before which the product should be consumed (expiration date)? | 🞏 Yes 🞏 No | NO |
| 1. Does it include a statement of the superiority of exclusive breastfeeding for the first six months and sustained breastfeeding until the child reaches two years? | 🞏 Yes 🞏 No | NO |
| 1. Does it include instructions for the appropriate preparation and use of the product? | 🞏 Yes 🞏 No | NO |
| 1. Does it include the words "Important Notice in Sub-Decree 133” of the health hazards of inappropriate use of the product? | 🞏 Yes 🞏 No | NO |
| 1. Does it contain a warning against the health hazards of inappropriate preparation and usage before a child reaches the recommended age? | 🞏 Yes 🞏 No | NO |
| 1. Does it contain a statement that the product should be used only on the advice of a health worker? | 🞏 Yes 🞏 No | NO |
| 1. Does it contain a statement that cup feeding is more hygienic than bottle-feeding? | 🞏 Yes 🞏 No | NO |
| 1. Does it contain text that may idealize the use of breast-milk substitutes, or discourage/undermine breastfeeding?   *Specify text*________________________________________________  *__________________________________________________________* | 🞏 Yes 🞏 No | YES |
| 1. Does it contain any images (photo, picture or graphic) other than those presenting the method of preparation or identifying the product as a breast-milk substitutes?   *Describe image (take photo):__________________________________* | 🞏 Yes 🞏 No | YES |
| 1. Does it contain information that implies or creates a belief that the product is used to replace/substitute breast-milk.   *Specify the information:*______________________________________  *__________________________________________________________* | 🞏 Yes 🞏 No | YES |
| 1. Does it contain a statement indicating the total cost of feeding for the first six months? *(only for infant formula for children less than 6 months)* | 🞏 Yes 🞏 No | NO |
| ***Additional statements of potential violation for similar products***  ***(sweetened condensed or skimmed milk, or similar products*** | | |
| 1. Does it contain a warning that this product shall not be used to feed infants and young children? | 🞏 Yes 🞏 No | NO |
| ***Additional statements of potential violation for complementary foods*** | | |
| 1. Does it contain instructions that the product should not be given to infants under 6 months? | 🞏 Yes 🞏 No | NO |
| 1. Does it contain text or images suggesting that the product should be given to infants under 6 months? *Specify:*___________________________________________________ | 🞏 Yes 🞏 No | YES |
| 1. Does it suggest that a feeding bottle should be used with this product? *Specify:*___________________________________________________ | 🞏 Yes 🞏 No | YES |
| ***Statements of potential violation for feeding bottles, teats and pacifiers*** | | |
| 1. Does it contain a statement of the superiority of breastfeeding for infant and young child feeding? | 🞏 Yes 🞏 No | NO |
| 1. Does it contain images or text that idealize the use of feeding bottles and teats?   *Specify:* ______________________________________________ | 🞏 Yes 🞏 No | YES |
| 1. Does it contain a statement that cup-feeding is safer than bottle-feeding?   *Specify*:______________________________________________ | 🞏 Yes 🞏 No | NO |
| 1. Does it contain instructions for appropriate cleaning of *feeding bottles/teats/pacifiers*? | 🞏 Yes 🞏 No | NO |

***REMINDER: AT THE END OF THE VISIT GIVE FEEDBACK ON THE RESULTS OF THE MONITORING TO THE OWNER/MANAGER/PERSON RESPONSIBLE FOR THE STORE/SHOP/PHARMACY***

| Date…………………………………………………………….. | Date………………………………………………………..………….. |
| --- | --- |
| Signature……………………………………………………… | Signature………………………………………………………..…… |
| Name of owner, manager, person responsible  …………………………………………………………………….. | Inspector name  ……………………………………………………..…………………… |
