# Supplementary material for: Pilot implementation of a monitoring and enforcement system for the International Code of Marketing of Breast‐milk Substitutes in Cambodia
Source: Matern Child Nutr. 2019 Jun 21;15(Suppl 4):e12795. doi: 10.1111/mcn.12795 (PMC6618142; doi:10.1111/mcn.12795)
Supplement: Supplementary file 2 — Data S2. Pilot checklist for monitoring promotions at the point‐of‐sale [file MCN-15-e12795-s002.docx]

Appendix II: Pilot checklist for monitoring promotions at the point-of-sale

**CHECKLIST FOR MONITORING AT THE POINT OF SALE**

| **General Information** | | | | | | |
| --- | --- | --- | --- | --- | --- | --- |
| Name of Province: | | | | | |  |
| Name of City/District: | | | | | |  |
| Name of Commune: | | | | | |  |
| Name of Store/Shop/Pharmacy/place: | | | | | |  |
| **Type of Promotion** | **Check if present** | **With MOH approval** | **Action** | **Company Name** | **Brand Name** |  |
| 1. Discount promotions   (e.g. coupon, stamps, discounts, special discount sales, premiums) | 🞏 | 🞏 Yes  🞏 No | **If you checked NO approval from MOH, report immediately**  **If APPROVED, VERIFY if any of the following are present**  **(If any CHECKED then REPORT)**  🞏 Health and nutrition claims that suggest the effects of the product on the child  🞏 Text/images that may idealize the use of breast-milk substitutes, or discourage/ undermine breastfeeding  🞏 Information that implies or creates a belief that the product is used to replace/substitute breast-milk |  |  |  |
| 1. Displays   (e.g. brand shelf, special displays, shop window, posters/banners, shelf tags/talkers, product launch, booth) | 🞏 | 🞏 Yes  🞏 No | **If you checked NO approval from MOH, report immediately**  **If APPROVED, VERIFY if any of the following are present**  **(If any CHECKED then REPORT)**  🞏 Health and nutrition claims that suggest the effects of the product on the child  🞏 Text/images that may idealize the use of breast-milk substitutes, or discourage/ undermine breastfeeding  🞏 Information that implies or creates a belief that the product is used to replace/substitute breast-milk |  |  |  |
| 1. Information materials   (e.g. pamphlets, booklets, leaflets) | 🞏 | 🞏 Yes  🞏 No | **If you checked NO approval from MOH, report immediately**  **If APPROVED, VERIFY if any of the following are present**  **(If any CHECKED then REPORT)**  🞏 Health and nutrition claims that suggest the effects of the product on the child  🞏 Text/images that may idealize the use of breast-milk substitutes, or discourage/ undermine breastfeeding  🞏 Information that implies or creates a belief that the product is used to replace/substitute breast-milk |  |  |  |
| 1. Free gifts or prizes | 🞏 | 🞏 Yes  🞏 No | **If you checked NO approval from MOH, report immediately.**  **I If APPROVED, VERIFY if any of the following are present (if any is CHECKED then REPORT)**  🞏 Health and nutrition claims that suggest the effects of the product on the child  🞏 Text/images that may idealize the use of breast-milk substitutes, or discourage/ undermine breastfeeding  🞏 Information that implies or creates a belief that the product is used to replace/substitute breast-milk |  |  |  |
| 1. Product samples | 🞏 | 🞏 Yes  🞏 No | **If you checked NO approval from MOH, report immediately.**  **If APPROVED, VERIFY if any of the following are present (if any is CHECKED then REPORT)**  🞏 Health and nutrition claims that suggest the effects of the product on the child  🞏 Text/images that may idealize the use of breast-milk substitutes, or discourage/ undermine breastfeeding  🞏 Information that implies or creates a belief that the product is used to replace/substitute breast-milk |  |  |  |
| 1. Company representatives/ promoters | 🞏 | 🞏 Yes  🞏 No | **If you checked NO approval from MOH, report immediately.**  **If APPROVED, VERIFY if any of the following are present (if any is CHECKED then REPORT)**  🞏 Health and nutrition claims that suggest the effects of the product on the child  🞏 Text/images that may idealize the use of breast-milk substitutes, or discourage/ undermine breastfeeding  🞏 Information that implies or creates a belief that the product is used to replace/substitute breast-milk |  |  |  |
| 1. Cross-Promotion   (e.g. product not covered by the Sub-Decree used to promote product covered by the Sub-Decree) | 🞏 | 🞏 Yes  🞏 No | **If you checked NO approval from MOH, report immediately.**  **If APPROVED, VERIFY if any of the following are present (if any is CHECKED then REPORT)**  🞏 Health and nutrition claims that suggest the effects of the product on the child  🞏 Text/images that may idealize the use of breast-milk substitutes, or discourage/ undermine breastfeeding  🞏 Information that implies or creates a belief that the product is used to replace/substitute breast-milk |  |  |  |
| 1. Tie-ins   (e.g. buy a Sub-Decree product and given extra product as a gift) | 🞏 | 🞏 Yes  🞏 No | **If you checked NO approval from MOH, report immediately.**  **If APPROVED, VERIFY if any of the following are present (if any is CHECKED then REPORT)**  🞏 Health and nutrition claims that suggest the effects of the product on the child  🞏 Text/images that may idealize the use of breast-milk substitutes, or discourage/ undermine breastfeeding  🞏 Information that implies or creates a belief that the product is used to replace/substitute breast-milk |  |  |  |
| 1. Other (*SPECIFY*): ______________   ______________ | 🞏 | 🞏 Yes  🞏 No | **If you checked NO approval from MOH, report immediately.**  **If APPROVED, VERIFY if any of the following are present (if any is CHECKED then REPORT)**  🞏 Health and nutrition claims that suggest the effects of the product on the child  🞏 Text/images that may idealize the use of breast-milk substitutes, or discourage/ undermine breastfeeding  🞏 Information that implies or creates a belief that the product is used to replace/substitute breast-milk |  |  |  |
| 1. Other (*SPECIFY*): ______________   ______________ | 🞏 | 🞏 Yes  🞏 No | **If you checked NO approval from MOH, report immediately.**  **If APPROVED, VERIFY if any of the following are present (if any is CHECKED then REPORT)**  🞏 Health and nutrition claims that suggest the effects of the product on the child  🞏 Text/images that may idealize the use of breast-milk substitutes, or discourage/ undermine breastfeeding  🞏 Information that implies or creates a belief that the product is used to replace/substitute breast-milk |  |  |  |
| 1. Other (*SPECIFY*): ______________   ______________ | 🞏 | 🞏 Yes  🞏 No | **If you checked NO approval from MOH, report immediately.**  **If APPROVED, VERIFY if any of the following are present (if any is CHECKED then REPORT)**  🞏 Health and nutrition claims that suggest the effects of the product on the child  🞏 Text/images that may idealize the use of breast-milk substitutes, or discourage/ undermine breastfeeding  🞏 Information that implies or creates a belief that the product is used to replace/substitute breast-milk |  |  |  |

***REMINDER: AT THE END OF THE VISIT GIVE FEEDBACK ON THE RESULTS OF THE MONITORING TO THE OWNER/MANAGER/PERSON RESPONSIBLE FOR THE STORE/SHOP/PHARMACY***

| Date…………………………………………………………….. | Date………………………………………………………..………….. |
| --- | --- |
| Signature……………………………………………………… | Signature………………………………………………………..…… |
| Name of owner, manager, person responsible  …………………………………………………………………….. | Inspector name  ……………………………………………………..…………………… |
