# Supplementary material for: Pilot implementation of a monitoring and enforcement system for the International Code of Marketing of Breast‐milk Substitutes in Cambodia
Source: Matern Child Nutr. 2019 Jun 21;15(Suppl 4):e12795. doi: 10.1111/mcn.12795 (PMC6618142; doi:10.1111/mcn.12795)
Supplement: Supplementary file 3 — Data S3. Pilot checklist for monitoring at health facilities [file MCN-15-e12795-s003.docx]

Appendix III: Pilot checklist for monitoring at health facilities

**PROMOTIONAL, INFORMATIONAL, AND EDUCATION MATERIALS CHECKLIST**

**FOR MONITORING AT THE HEALTH FACILITY**

| ***General Information*** | |
| --- | --- |
| **Province/Municipality** | ___________________________________ |
| **Operational District** | ___________________________________ |
| **Type of health facility** | 🞏 Health Centre ………….………….……………….  🞏 Private Clinic ……………..…………….………….  🞏 Hospital ….………………..………………..……….  🞏 Other (*specify*) ……………………………….…… |

| **Content** | **Can you see any of the following?**  **(please check what you see)** | **if checked:** | | |
| --- | --- | --- | --- | --- |
|  |  | **write**  **Company Name** | **write**  **brand name** | **action** |
| 1. Equipment showing company brands or logos | 🞏 Clocks |  |  | take sample/ photo & report |
|  | 🞏 Tables |  |  | take sample/ photo & report |
|  | 🞏 White board |  |  | take sample/ photo & report |
|  | 🞏 Chairs |  |  | take sample/ photo & report |
|  | 🞏 Copy machines |  |  | take sample/ photo & report |
|  | 🞏 Other, *specify:*___________ |  |  | take sample/ photo & report |
|  | 🞏 Other, *specify:*___________ |  |  | take sample/ photo & report |
|  | 🞏 Other, *specify:*___________ |  |  | take sample/ photo & report |
| 1. Other promotional materials | 🞏 Pens |  |  | take sample/ photo & report |
|  | 🞏 Calendars |  |  | take sample/ photo & report |
|  | 🞏 Posters |  |  | take sample/ photo & report |
|  | 🞏 Note pads |  |  | take sample/ photo & report |
|  | 🞏 Toys |  |  | take sample/ photo & report |
|  | 🞏 Growth charts |  |  | take sample/ photo & report |
|  | 🞏 Clothing/T-shirt/Baby Bib |  |  | take sample/ photo & report |
|  | 🞏 Shelf talker |  |  | take sample/ photo & report |
|  | 🞏 Other, *specify:*___________ |  |  | take sample/ photo & report |
|  | 🞏 Other, *specify:*___________ |  |  | take sample/ photo & report |
|  | 🞏 Other, *specify:*___________ |  |  | take sample/ photo & report |
| 1. Informational or educational materials with company logos or brands | 🞏 Brochure |  |  | take sample/ photo & report |
|  | 🞏 Booklet |  |  | take sample/ photo & report |
|  | 🞏 Informational poster |  |  | take sample/ photo & report |
|  | 🞏 Video |  |  | take sample/ photo & report |
|  | 🞏 Other, *specify:*___________ |  |  | take sample/ photo & report |
|  | 🞏 Other, *specify:*___________ |  |  | take sample/ photo & report |
|  | 🞏 Other, *specify:*___________ |  |  | take sample/ photo & report |

| **Product sale, sample, display and company visits** | **Answer** | **If Yes Checked:** | | |
| --- | --- | --- | --- | --- |
|  |  | **write**  **Company Name** | **write**  **brand name** | **action** |
| 1. Are designated products being sold inside the health facility? | 🞏 Yes  🞏 No |  |  | take photo if possible & report |
| 1. Are designated products displayed inside the health facility? | 🞏 Yes  🞏 No |  |  | take photo if possible & report |
| 1. Do you see any invitations to attend meetings, parties, and events for the health workers and/or clients? | 🞏 Yes  🞏 No |  |  | take photo if possible & report |
| 1. Are samples of designated products available in the Health Facility? | 🞏 Yes  🞏 No |  |  | take photo if possible & report |
| 1. Are there company representatives/ promoters talking to pregnant women, mothers, caregivers and families? | 🞏 Yes  🞏 No |  |  | take photo if possible & report |

| **Questions for pregnant women and mothers** | **Answer** | **If Yes Checked:** | | |
| --- | --- | --- | --- | --- |
|  |  | **write**  **Company Name** | **write**  **brand name** | **action** |
| 1. In the past month, have you ever received a **gift** from milk company representatives while you were in this health facility? | 🞏 Yes  🞏 No |  |  | report |
| 1. In the past month, have you received free **samples** of any milk/food products for children 0-24 months in this health facility? | 🞏 Yes  🞏 No |  |  | report |
| 1. In the past month, did any company representative/ promoter **talk** to you/ invited you to an event/ party/join a contest while you were inside this health facility? | 🞏 Yes  🞏 No |  |  | report |
| 1. In the past month did a health worker **recommend** milk/food products for children 0-24 months? | 🞏 Yes  🞏 No |  |  | report |

| **Questions for health workers** | **Answer** | **If Yes Checked:** | | |
| --- | --- | --- | --- | --- |
|  |  | **write**  **Company Name** | **write**  **brand name** | **action** |
| 1. In the past month, have you ever received a **gift** from milk company representatives? | 🞏 Yes  🞏 No |  |  | report |
| 1. In the past month, have you received free **samples** of any milk/food products for children 0-24 months? | 🞏 Yes  🞏 No |  |  | report |
| 1. In the past month, did any company representative/promoter **talk** to you/ invited you to an event/ party/join a contest while you were inside this health facility? | 🞏 Yes  🞏 No |  |  | report |

***REMINDER: AT THE END OF THE VISIT INFORM THE HEAD OF THE FACILITY OR HIS/HER REPRESENTATIVE OF THE FINDINGS OF THE MONITORING.***

| Date…………………………………………………………….. | Date………………………………………………………..………….. |
| --- | --- |
| Signature……………………………………………………… | Signature………………………………………………………..…… |
| Name of head of facility/representative  …………………………………………………………………….. | Inspector name  ……………………………………………………..…………………… |
