# Supplementary material for: Pilot implementation of a monitoring and enforcement system for the International Code of Marketing of Breast‐milk Substitutes in Cambodia
Source: Matern Child Nutr. 2019 Jun 21;15(Suppl 4):e12795. doi: 10.1111/mcn.12795 (PMC6618142; doi:10.1111/mcn.12795)
Supplement: Supplementary file 4 — Data S4. Pilot Violation Reporting Form [file MCN-15-e12795-s004.docx]

Appendix IV: Pilot Violation Reporting Form

**Annex 5: Monitoring reporting form of violation**

Pursuant to Article 17 (c) of the Sub-Decree on Marketing of Products for Infant and Young Child Feeding and Article 7 (3) of the Joint Prakas on implementation thereof.

If you notice any promotion by baby food companies or any person who works for or in the interest of such companies, please complete the form below, send it together with your examples or photos or pictures (if any) to **The Secretariat of the Executive Working Group, Sub-Decree on Marketing of Products for Infant and Young Child Feeding, c/o Ministry of Health.**

**Description of Violation**

1. **Short Description:**

……………………………………………………………………….……………………………………………………….………………………………………… *(Describe the promotion and include heading or slogan found on promotional materials, for example: newspaper advertisement: “Bringing up Champion “or “Gift bags to mothers upon discharge from hospital”)*

1. **When was the violation observed:** (dd/mm/yyyy and what time) ………………………….…………….…..……..………………
2. **Where** (Place, town, others)………………………………………………..……………………………………..……………….………………….…

*(For newspapers and periodicals, please indicate the name and date of publication, for TV/Radio indicate TV channel, Radio frequency, from website/Facebook: web page or Facebook account)*

1. **Company Name:** ……………………………………………………..……………………………………………………………………………………….
2. **Brand Name:** ………………………………………..……………………………………………………………………………………..…………………..
3. **Type of product being promoted:** Please indicate the relevant item by ticking (✓) the box on the right.

| Infant Formula including special formula (0-6 months) |  | Follow up Formula  (6 months and above) |  | Complementary Food (*Please describe*)  ……………………………… |  | Liquid product such as bottled water, tea or juice marketed for infants and young children |  |
| --- | --- | --- | --- | --- | --- | --- | --- |
| Feeding bottles |  | Teats |  | Pacifiers |  | Other product  (*Please describe)*  ……………………………… |  |

1. **Type of violation:** Please indicate the relevant type by ticking (✓) the box on the right.

| Advertisement |  | Promotion in shops |  | Free sample |  | Donation of non-scientific and factual materials |  | Promotion in health facilities |  |
| --- | --- | --- | --- | --- | --- | --- | --- | --- | --- |
| Gift to health workers or association |  | Events/gifts targeting mothers, etc. |  | Sponsorship (events, study, research), salary service, etc. |  | Work benefits, sales quota |  | Inadequate labeling |  |

Name: ……………………………………………………………………………….………………………………………

Address: ………………………………………………………………………………….………………………………..

| The above information is necessary to enable the Oversight Board to double-check the information you have given, if necessary. If you wish to keep your identity confidential, remember to tick (✓) this box: 🞏 |
| --- |

Contact Number: ………………………………………………………………………………………………………
